# Threat to Freedom and the Detrimental Effect of Avoidance Goal Frames: Reactance as a Mediating Variable

**DOI:** 10.3389/fpsyg.2016.00632

**Published:** 2016-05-18

**Authors:** Daniela Niesta Kayser, Verena Graupmann, James W. Fryer, Dieter Frey

**Affiliations:** ^1^Division of Social Psychology, University of PotsdamPotsdam, Germany; ^2^Department of Psychology, DePaul University, ChicagoIL, USA; ^3^Department of Psychology, State University of New York at Potsdam, PotsdamNY, USA; ^4^Department of Social Psychology, Ludwig Maximilian University of MunichMunich, Germany

**Keywords:** freedom restriction, goal frames, avoidance, approach, reactance, self threat, change

## Abstract

Two experiments examined how individuals respond to a restriction presented within an approach versus an avoidance frame. In Study 1, working on a problem-solving task, participants were initially free to choose their strategy, but for a second task were told to change their strategy. The message to change was embedded in either an approach or avoidance frame. When confronted with an avoidance compared to an approach frame, the participants’ reactance toward the request was greater and, in turn, led to impaired performance. The role of reactance as a response to threat to freedom was explicitly examined in Study 2, in which participants evaluated a potential change in policy affecting their program of study herein explicitly varying whether a restriction was present or absent and whether the message was embedded in an approach versus avoidance frame. When communicated with an avoidance frame and as a restriction, participants showed the highest resistance in terms of reactance, message agreement and evaluation of the communicator. The difference in agreement with the change was mediated by reactance only when a restriction was present. Overall, avoidance goal frames were associated with more resistance to change on different levels of experience (reactance, performance, and person perception). Reactance mediated the effect of goal frame on other outcomes only when a restriction was present.

## Introduction

The antagonism between the inevitability of change and resistance to change is deeply ingrained in human thinking and acting. A general aversion to change has been attributed to a fundamental motivation to favor previously made choices and to attach ourselves to courses of action, once they are initiated (Cialdini et al., 1995). In everyday life, we often encounter demands for change of our behavior, e.g., adapting the syllabus of a research methods course to follow new department guidelines. The motivation and likelihood with which we are going to implement the requested change to our syllabus depends upon the degree to which we perceive such a demand as a threat to our freedom. Our inclination to make the change may also be influenced by whether the demand has been communicated as aimed at achieving better learning outcomes or avoiding negative learning outcomes. Does communication style additionally influence the perceived threat to freedom in the request? And if so, are there requests that are more restricting than others? The present experiments were designed to examine whether and how communication style framed with approach and avoidance goals affects the impact of freedom restrictions.

### Change and Psychological Reactance

Within the field of social psychology the concept of freedom is mostly looked at in the context of the individual’s control and choice. Reactance theory (Brehm and Brehm, 1981) in particular emphasizes the importance of individual freedom and behavioral choices and defines conditions under which people react against attempts to control their behavior and eliminate their freedom of choice. Freedom in terms of reactance theory is defined as a person’s belief to be able to engage in a certain behavior and to decide on the type of behavior, as well as how the behavior is performed and when. Thus, reactance theory proposes that when we believe to be free to choose a course of action, we experience reactance if that freedom is eliminated or threatened. According to Brehm (1966), psychological reactance is an aversive motivational state directed toward the re-establishment of freedom, even if that resistance is not associated with optimal outcomes for the person. The potentially negative consequences of reactance are mirrored in the fact that the term is adapted from the field of electrical engineering and means ‘blind resistance.’ Reactance manifests itself in an increased desire to engage in the restricted behavior or actual attempts to engage in it, or an active refusal to engage in the prescribed behavior, e.g., students may decrease their own effort, patients may not adhere to treatment plans, or employees may resist the implementation of a new strategy. When Amy asks her friend Rachel to accompany her to the concert of their favorite band and uses the message “you must come with me,” reactance is likely aroused by forcing a desired outcome on the person; likewise, reactance is aroused as well by eliminating access to a desired outcome such as when parents forbid their daughter to attend the concert: “you must not go to this concert” (Wicklund, 1974). Where freedom is threatened by social pressure, reactance evokes the tendency to reassert the lost freedom by the individual to resist that pressure, e.g., by reducing one owns efforts to slow down the proposed goal or by disagreeing with the communicator to weaken the implementation of a project (e.g., Dillard and Shen, 2005; Silvia, 2006). Reactance occurs in response to a message that implies a forceful attitude and makes the person want to approach a desired goal, which will lead the person to resist this *imposition* or the way the request is *imposed* upon them. Likewise, reactance occurs when the message contains a perceived threat and prohibits a specific behavior or demands the person to *stay away* from a desired goal. This will likely lead the person to resist this *prohibition* or the way the *prohibition* is phrased.

In its origin, reactance theory examined how people can be persuaded, attitudes can be changed, and consumer behavior can be influenced. The theory extends to other aspects of individual behavior that involve motivation following uncontrollable events, in particular, achievement motivation and *task performance*. In contrast to Seligman’s (1975) theory of learned helplessness, which predicts a decrease in subsequent motivation and performance a loss of control over outcomes is experienced, whether or not it is aversive, reactance theory predicts increased motivation and performance. In order to accommodate these seemingly opposing predictions, Wortman and Brehm (1975), proposed an integrative model: When perceived control over an outcome is high, psychological reactance results in enhanced motivation to achieve and perform well when having to overcome resistance. However, when participants’ expectation of control is low, subsequent performance should deteriorate when facing resistance. In line with this model and based on the work by Folkman and Lazarus (1985), experienced stress induced for example by particular demands of a task, can be evaluated as challenging or threatening. Perceptions of challenge enhance performance, whereas perceptions of threat inhibit performance. Moreover, introducing change in the rules of the task at hand has shown to have a negative effect on participants’ performance. In a study by Drach-Zahavy and Erez (2002), threat was operationalized in terms of negative outcomes and an interview with the five worst performers, a procedure that set a focus on failure (as opposed to the challenge condition, where the focus was set on success). This procedure deemed to be particularly detrimental in a change situation in which the participants had to use a different strategy at round 2 compared to round 1.

Particularly owing to the work on stereotype threat, the link between existence of a threat and depletion of self and lowered performance (e.g., Baumeister, 1984; Steele and Aronson, 1995; Frederickson et al., 1998) is well documented. Similarly, a threat induced by a demand to change should consume resources, divide attention away from the task, and therefore disrupt and diminish an individual’s mental activity. A change request in the academic context, we argue, can be experienced as a threat, and if it is, it should impair intellectual performance.

Reflected in the current literature on social influence, requests to change are especially pertinent to questions of *persuasive communication*. It might represent a threat to Rachel, when Amy communicates the message that they ‘must’ visit a concert of a band that they both like, even if Rachel is a fan of this band herself. In fact, one of the basic claims of reactance theory is that high-pressure communicators are likely to be seen as threatening to personal freedom (Wicklund, 1974; Brehm and Brehm, 1981). In a study requesting the change for the use of washing detergents from containing phosphates to not containing phosphate, one indication of consumers’ reluctance to participate in this change of habit was expressed in their beliefs that washing detergents without phosphate would be less efficient (Mazis et al., 1973).

Research examining the role of state-(Rains and Turner, 2007) trait-(Pavey and Sparks, 2009) reactance in how persuasive information on health risks is evaluated and affects intentions to engage in health behaviors has shown a negative relationship between reactance and the intended effect of such messages. Here reactance has also been associated with a perception of threatened freedom, further supporting the original theory in the domain of persuasion.

Derogating the *object* is one possible avenue to restore freedom, while derogating the *source of threat* (Kohn and Barnes, 1977) is another possible outcome of reactance albeit indirectly. This is consistent with the initial theoretical reasoning by Brehm (1966) arguing that reactance could not be measured directly, but instead be inferred by its effects. Since the beginning of the work on reactance, reactance has been implicitly and explicitly defined in many different ways. Among other accounts, reactance has been viewed as *cognitive* (e.g., Petty and Cacioppo, 1986), i.e., measurable through thoughts that can be listed in self-report techniques and operationalized in terms of counter-arguing. Since reactance evokes anger and is associated with the experience of hostile feelings (Wicklund, 1974), it has also been considered an *emotion* (e.g., Dillard and Mejenders, 2002).

Finally, whether the motivation to restore freedom following an uncontrollable event is set in the domain of achievement or persuasion, reactance as a motivated psychological state will likely guide attention, influence thought process (Derryberry and Tucker, 1994), stimulate feelings, and direct behavior (Kuhl, 1986). Change induced in an aversive manner may therefore be reflected in reactance shown in cognitive, affective, and behavioral outcomes.

### Change and Goal Framing

Change implies a new direction and new goals that can imply the elimination of options and the reduction of a set of perceived freedoms. A restricted freedom may yield greater aversion when pursued with a certain type of goal. Avoidance goal frames focus on trying to avoid or stay away from a negative outcome or a negative psychological situation. Examples of avoidance goal frames are “Try to avoid doing poorly on a test,” “Try not to be disloyal to friend,” and “Try to avoid smoking a cigarette.” Approach goals, on the other hand, use positive, desired possibilities such as “Try to do well on a test,” “Try to be a loyal friend,” and “Try to become smoke-free,” which typically leads to favorable psychological processes and outcomes, such as perceptions of personal progresses or competence in goal pursuit (Elliot and Sheldon, 1997; Elliot et al., 1997). The approach-avoidance distinction is integral to the understanding of how individuals deal with changes, as requests to change always also imply a change in motivational tendency to initiate a shift in a person’s course of action. The type of goal that is pursued in light of a restriction leads to a number of important implications for the experience of threat.

### Goal Framing and Reactance

Looking at goal framing and how it relates to a person’s sense of freedom and threat to freedom, the experience of the different goal frames in terms of reactance should vary according to the respective goal frame: Approach goal frames suggest a change in course of action that can be a potential gain to the status quo, i.e., no change in course of action will lead to a neutral outcome, whereas a change leads to a positive outcome, and therefore change can be interpreted as optional. In this vein, a sense of behavioral freedom is maintained and approach goal frames associated with a restricted freedom should therefore yield less psychological reactance.

Avoidance goal frames, on the other hand, suggest a change in course of action that is required in order to avoid experiencing a deterioration of the status quo, i.e., no change in course of action will lead to a negative outcome, and therefore the change is not optional. Therefore setting an avoidance goal frame when told to change has the potential to increase the perception of restriction to behavioral freedom. A change embedded in an avoidance goal frame might thus be associated with more psychological reactance than when embedded in an approach goal frame. Moreover, the inherent focus on negative possibilities in avoidance goal frames has shown to be associated with a host of aversive psychological processes, including perceptual, attentional, mental control, emotional and behavioral processes, for example experiencing fear of failure, anxiety, and wanting to escape from the goal-relevant situation (for example, Wegner, 1994; Elliot and McGregor, 1999). This link between avoidance goal frames and aversive psychological processes can be interpreted as a threat to a central self-motive (Graupmann et al., 2013), which may result, among others in a tendency to react against the implied restriction of freedom.

Research has yet to directly examine the link between the restriction of a freedom, avoidance goal pursuit and the emergence of reactance. If freedom was threatened by implication, the emergence of reactance when avoidance but not approach goal frames are salient would suggest that this type of goal framing is experienced as a self-threat. Along with this idea, research on the self posits that self-regulation draws on a limited common pool of resources (Baumeister, 1998). Since regulating the self is difficult, subsequent acts of self-regulation entail a state of ego-depletion (Baumeister, 1998). An individual that engages in goal-directed behavior and that monitors his/her goal progress expends resources. Furthermore, research on goal pursuit documents that the pursuit of some types of goals is more depleting than others (Oertig et al., 2013). In particular, avoidance compared with approach goal frames have been shown to deplete self-regulatory resources, which is manifested in perceptual, attentional, emotional, and behavioral deficits. This grounding in negative possibilities may produce aversive psychological processes that have negative consequences such as resistance, impairment of performance, and disagreement with social influence; processes much as experienced following a perceived elimination of freedom.

According to this goal-pursuit-reactance approach to self-threat, an avoidance frame in a restricting situation should not only be detrimental to the willingness to comply with the request, but also be more self-impairing than an approach frame: avoidance goal frames have been repeatedly found to lead to a less deep processing of tasks, to preparing things in a more disorganized fashion, to worrying more about the own competence and to elicit more negative emotional reactions (Elliot and McGregor, 1999).

People are generally motivated to approach positive outcomes and avoid negative outcomes. However, to the best of our knowledge, only limited research has been conducted on the interplay of the impact of goal framing on performance and perception processes when a freedom is restricted. Integrating predictions from reactance theory and approach avoidance accounts into a larger theoretical framework enables us to test the impact of an avoidance goal frame when a person is told to change and, more importantly, through the experience of reactance of this person and her subsequent task performance and person perception processes.

Combining these two lines, perceptions of and requests to change are a threat due to (1) the potential to restrict one’s freedom as predicted by reactance theory and (2) the threatening nature when the change is framed in terms of avoidance goals.

### Overview of the Present Research

In the present research, we investigate the link between goal framing and psychological reactance as an indication of threat to self. On the basis of both theory and prior empirical work on goals and resistance to change, we hypothesize that change requests presented with an avoidance goal frame will be associated with (a) worse performance and (b) more negative evaluations of competence (own and communicator’s) than change requests presented with approach goal frames. We further hypothesize that change requests presented with an avoidance (as compared with approach) goal frame will be associated with (c) more experience of reactance and in turn negatively affect the outcome. We have conducted two studies designed to test this set of hypotheses. In Study 1, a change request implied a restriction by asking participants to switch from a preferred working strategy to a new one that they previously did not chose and the goal frame was varied between approach and avoidance. In Study 2, we systematically varied the presence of a freedom restriction, as well as the goal frame in a message given to student participants about a proposed change in their study program. In both studies all procedures were in accordance with the local IRB regulations and the Declaration of Helsinki.

## Study 1

A goal is a cognitive representation of a possible state or outcome that an individual seeks to attain (Austin and Vancouver, 1996; Elliot and Thrash, 2002) and goals focus on either a positive or a negative possibility. Given that the way in which a goal is worded corresponds to the way in which the goal is represented in memory (Elliot and Friedman, 2007), we systematically varied the frame of the goal in which the change request was phrased.

### Method

We conceptualized a change request presented as a restriction framed with an approach goal as something that the participants “must do” versus a restriction framed with an avoidance goal as something that the participants “must not do.”

With the goal of 50 participants, we began collecting data from a college student sample during the spring semester and terminated data collection when the academic year ended leaving a final *N* = 56.

#### Participants and Design

Fifty-six (39 women) undergraduates at the University of Rochester participated in the experiment entitled “Applied performance and problem solving” in return for course credit. The mean age of participants was 20.00 years (range = 18–24). Three participants who failed to engage in the puzzle-solving task were excluded from the analyses. Participants were randomly assigned to one of two between-subjects goal frame conditions: the approach goal frame condition (*n* = 29) or the avoidance goal frame condition (*n* = 24). The approach goal frame condition was manipulated by the wording “For the following task, you must choose a different strategy than the one that you just used” in half of the cases. The avoidance goal frame condition was manipulated by the wording “For the following task, you must not choose the same strategy that you just used” in the other half. The experimenters in this and the subsequent experiment were blind to participants’ condition, and remained unaware of the hypotheses being tested throughout data collection.

#### Procedure

Upon arrival for the experiment, participants were greeted and presented with a computer-based problem-solving task on a screen (adopted from Förster et al., 1998, Study 3; anagram test). The screen displayed a collection of the letters d, p, q, b, r, and g presented in a rectangular box with rows and columns filled with letters where each letter was presented exactly 114 times^1^. The participants received the written instruction that completing the task consisted of counting how many times the letter p was presented on the screen and that they were under no time limit.

Following the presentation of the letters with an open time frame, participants were free to choose one among four possible strategies to complete the initial task for practice purposes: One strategy involved searching letters row by row, a second one involved searching letters column by column, a third involved searching letters grid by grid and the fourth involved searching the screen as a whole. After these strategies were explained to participants, they selected the strategy that suited them the most. They completed the task again with no time limit which consisted in counting how many times the letter p was presented and in entering the correct number in a field on the bottom of the screen online. After the participants finished solving the initial task, they were told to complete a second problem-solving task (very similar to the first task), hereby inducing the change. The change consisted in the explicit instruction to use a different search strategy than the one they had just adopted for the initial task, hereby manipulating the respective goal frame condition. This constituted the second round of solving the puzzle. The second task used the letters v, k, w, x, y, and z (to avoid habituation); participants were asked to search for the letter v (again same amount of letters for each letter category).

After completing the tasks, the participants were administered the measures and then given a final questionnaire assessing their basic demographics. Finally, participants were debriefed about the purpose of solving the puzzle and informed that the session was now over.

#### Measures

##### Accuracy of solving the puzzle

A difference measure of how many letters were correctly identified in each of the two rounds was calculated to represent the overall accuracy of solving the puzzle and the performance of the participants. Similar to previous studies in which the absolute difference scores were computed (see Drach-Zahavy and Erez, 2002: high difference scores represented low performance), we took the *absolute* number of correctly identified letters at round 1 and subtracted it from the *absolute* number of correctly identified letters at round 2. If the resulting number was positive, the participants correctly identified more letters at round 1 compared with round 2. Conversely, if the participants identified more letters at round 2 compared with round 1, the resulting number was negative. In this manner, we were able to determine the accuracy of solving the puzzle and the performance accounting for the change from round 1 to round 2. Hence, not the performance *per se* at time 2, but the difference between the two rounds contingent on the respective goal frame condition was measured. Unlike previous research, we did not assess stress appraisals of challenge as opposed to threat, but manipulated the threat by restricting the strategy that the participants could select for the second task.

##### Reactance

Experience of threat to freedom in the form of psychological reactance was assessed with nine items used in previous research (Jonas et al., 2009). Items include “How reasonable did the request to change the strategy appear to you?” and “How restricted did you feel in your freedom to choose the strategy you wanted to use”? (1 = not at all, 10 = extremely) Scores were averaged to form a composite index (α = 0.83).

##### Additional measures

Two one-item measures for *perceived task difficulty* (“How difficult do you think the task was”) and *perceived competence* (“How competent do you think you were in solving the puzzle”; 1 = not at all, 10 = extremely) were adapted from existing measures (Elliot and Church, 1997) for performance-avoidance goal frames.

### Results

#### Performance

A one-sample *t*-test on mean performance of participants between the two rounds showed that performance did not improve in general between round 1 (*M* = 7.90, *SD* = 6.49) and round 2 (*M* = 7.05, *SD* = 7.49), *t*(52) < 0.50, *p* > 0.64). Additionally, an independent sample *t*-test on performance in round 1 showed that it was similar in both goal framing conditions (Diff = -2.99, *SD* = 1.82), *t*(51) = -1.64, *p* = 0.11. Next, we tested whether performance differed as a function of condition and option selected by the participants at round 2. There were no significant main effects nor interaction, all *F*s < 1.62, all *p*s > 0.199.

**FIGURE 1:**
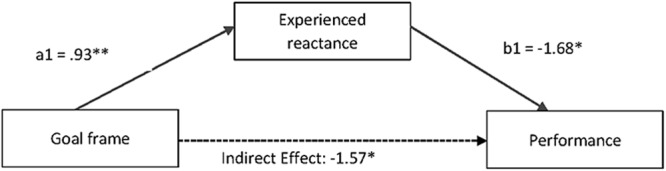
**Mediation model.** All path coefficients represent unstandardized regression weights. Because the two experiment groups are coded by a one unit difference, the total effect of 3.23 can be interpreted as a mean difference. Total adjusted *R*^2^ for the model = 0.11, *F*(2,50) = 3.18, *p* = 0.05. ^∗^*p* < 0.05, ^∗∗^*p* < 0.01.

According to our hypothesis, a change request in an avoidance frame corresponds to an increase in perceived freedom restriction (i.e., a threat) which translates into increased reactance which, in turn impairs performance. We tested this mediational hypothesis using the bootstrapping procedure and corresponding SPSS macro *process* of Hayes (2012) developed for mediation and moderation analysis. It allowed us to test for indirect effects, regressing performance onto goal frame (dummy-coded: approach = 0/avoidance = 1), with reactance as the proposed mediator. One thousand bootstrap resamples were performed. As expected, we found an overall effect for goal frame and reactance on performance, *R*^2^adj. = 0.11, *F*(2,50) = 3.18, *p* = 0.048. First, a direct effect of goal frame on performance shows that those who were asked with an avoidance frame (compared to an approach frame) to switch their strategy found 4.79 more letters at round 1 compared with round 2, *t*(51) = -2.07, *p* = 0.036, 1 - β = 0.708, with a 95% confidence interval excluding zero (0.146–9.441), hence doing worse than those asked with an approach framed goal. Moreover, those who were asked with an avoidance frame were 0.93 relatively more reactant, *t*(51) = 2.51, *p* = 0.015, 1 – β = 0.669, with a 95% confidence interval excluding zero (0.185–1.675) than those asked with an approach frame, a score resulting from the difference in reactance following a one-unit change in goal frame. Additionally, those who felt relatively more reactant found on average 1.68 less letters at round 2, *t*(51) = -2.04, *p* = 0.047, 1 - β = 0.712, with a 95% confidence interval excluding zero (-3.337 to -0.028). Most importantly, the indirect effect of goal frame on performance through reactance is negative and statistically different from zero as evidenced by a 95% bias-corrected bootstrap confidence interval that is entirely below zero (-4.11 to -0.19). Suggesting that those participants confronted with an avoidance framed change request performed 1.57 times worse on average than those confronted with an approach framed change request, as a result of the mediation by reactance (see **Figure 1**, for a graphical depiction of the mediation model).

#### Reactance and Additional Dependent Variables

First, we tested the impact of goal framing on reactance, task difficulty, and self-perceived competence to compare the differential effect of approach and avoidance goal framing. Avoidance as compared with approach frame increased reactance, *t*(51) = -2.51, *p* = 0.015, *d* = -0.67, but had no differential impact on task difficulty or competence, *t*s ≤-0.72, *p*s ≥ 0.48; see **Table 1** for means and standard deviations. Next, we used a moderated regression analysis to explore the relationship between reactance, task difficulty, and self-perceived competence moderated by condition. While reactance had an overall negative impact on task difficulty, β = 0.291, *t*(50) = 2.17, *p* = 0.035, it had no effect on competence, β = 0.168, *t*(50) = 1.22, *p* = 0.227, and no effect on either outcome variable when moderated by condition^2^.

**Table 1:** Means and standard deviations for reactance, task difficulty and self-perceived competence in Study 1.

	Reactance		Task difficulty		Self-perceived competence	
Goal frame	*M*	*SD*	*M*	*SD*	*M*	*SD*
Approach goal frame	2.96	1.35	4.07	1.44	4.33	1.03
Avoidance goal frame	3.89	1.35	4.13	1.45	4.54	1.14

Finally, we tested whether task difficulty or competence mediated effects on performance, finding a mediation only for competence, *R*^2^ = 0.124, *F*(2,50) = 3.55 *p* = 0.036, showing that more competence valuation yields a smaller number of mistakes when searching for the correct letters (β = 0.296, *p* = 0.032)^3^.

### Discussion

In line with our hypotheses, when a change was communicated with an avoidance goal frame as compared to an approach goal frame, participants in Study 1 showed impaired task performance. They also indicated to experience more threat to freedom, i.e., psychological reactance, when avoidance compared to approach was the goal frame. Importantly, reactance mediated the effect of goal frame on the outcome: Avoidance (vs. approach) goal frame increased perceived threat to freedom, which in turn impaired task performance. This finding supports our theoretical claim that individuals who focus on a negatively phrased restriction or undesired option, here a *prohibition to do something*, face a greater aversive motivational state than individuals who focus on a positively phrased restriction or desired option, here an *order to do something*, even when the content of the task itself remains almost identical. This is also in line with previous research on approach and avoidance goal frames in the achievement literature which documents that individuals are more disorganized and challenged when they set avoidance framed performance goals (Elliot and McGregor, 1999). Only those participants in the avoidance (compared with the approach condition) who scored high in the perceived difficulty of the task showed a negative relationship between reactance and perceived competence. This finding resonates with empirical evidence on the effects of threat versus challenge in a task situation, in which a more difficult task (task difficulty perceived to be high) was more likely to produces a threat (Förster et al., 1998). For the purpose of our task, feedback was not mentioned and not given, so in neither of the conditions appraisal of own competence were possible.

Having obtained initial evidence that a perception of threat to freedom is associated with more negative outcomes in task performance when a change request is framed with an avoidance goal, we intended to more directly test the role of freedom restriction, introducing a condition that does not imply a restriction in Study 2. Here we examined the impact of goal frame in interaction with the presence versus absence of an actual restriction to freedom. Furthermore, we intended to see whether the effect of goal frame would replicate in a situation of persuasive influence, looking at communicator variables and persuasion in Study 2. Finally, we explored the effect of goal frame and restriction on affect.

## Study 2

The cognitive response approach (Petty et al., 1981) assumes that the impact of a message on attitudes is mediated by cognition. In hearing or reading a persuasive message, individuals generate cognitions that can be in agreement or disagreement with the message. Dillard and Shen (2005) contend that it is plausible that individuals respond to freedom-threatening messages with unfavorable cognitions about the message and about the communicator.

Accordingly, we expect to find that individuals confronted with a potential change presented with an avoidance compared to an approach frame will experience the avoidance frame as more threatening to their freedom which in turn will lead to a lower agreement with and more counterarguing regarding the proposed change. We further expect to find that individuals in the avoidance compared with the approach frame condition will evaluate the communicator of a threatening message more negatively. This negative evaluation will be shown on dimensions such as the perceived trustworthiness and competence of the communicator. We further expect to find that those individuals who are confronted with a restriction framed with an avoidance goal will experience the strongest reactance and that reactance will act as a mediator between the goal frame and the outcome, here the agreement with the proposed change. In Study 2, we also assessed differences in experienced emotions.

### Method

Our heuristic approach was to match participant numbers of previous studies on persuasive threat attempts and resistance to attitude change, which obtained medium to large effect sizes (e.g., Miller et al., 2007). With the goal of 100 participants, we began collecting data from a college student sample at a German university during the summer semester and terminated data collection when the academic year ended leaving a final *N* = 105.

#### Participants and Design

One hundred and five (79 women) participants volunteered toward the end of an undergraduate lecture to evaluate what was presented as the integration of a new program in return for course credit. The mean age of participants was 20.90 years (range = 19–43). Participants were randomly assigned to conditions in a two (restriction: yes vs. no) by two (goal frame: approach vs. avoidance) factorial design.

#### Procedure

First, the participants read a cover story in which a professor described and advertised a new concentration named ‘Urban Design’ as allegedly being implemented to the psychology program in the near future. They were informed that as a consequence of this addition a total of 13 instead of (now) 12 concentrations would be part of the program. The text varied between four scenarios, namely whether the addition would entail restrictions in terms of limited access to existing seminars (restriction present) versus no limitations (restriction absent) for the students and whether the addition of the new concentration was framed in terms of an approach or avoidance goal. In the approach goal frame conditions the change was described as helping to improve the university’s high ranking whereas in the avoidance goal frame condition the change was described as helping to avoid a decrease in the ranking.

After reading the text participants responded to questions regarding their perception of the message (message evaluation: agreement with change), communicator (communicator evaluation: trustworthiness, competence), cognitive response: counterarguing, their perceived threat to freedom (reactance) and their affective (positive, negative) state. Next, participants were given a final questionnaire assessing their basic demographics. Finally, participants were debriefed about the purpose of the survey’s content and informed that the session was now over.

#### Measures

##### Message evaluation

To assess participants’ *agreement with integration of new concentration* we used a five-item measure by Miller et al. (2007), e.g.: “*How much would you support the implementation of such a program.”* (α = 0.89).

##### Communicator evaluation

To assess participants’ perception of the communicator’s competence we used a three-item measure of *perceived competence of the communicator* (Dillard and Shen, 2005), e.g.: “*How qualified does the person who communicated the topic appear to you*.” (α = 0.91). To assess perceived *trustworthiness*, we used a two-item measure used in previous research on the impact of reactance on person perception (Silvia, 2006), e.g.: “How trustworthy does the person who communicates the topic appear to you?” (Spearman–Brown ρ = 0.88).

##### Cognitive response

To assess *counterarguing* we used a three-item measure adapted from Rains and Turner (2007), e.g., *“Did you develop counterarguments against the here presented position?* (α = 0.91).

##### Reactance

Threat to freedom in the form of psychological reactance was assessed exactly as in Study 1, and scores were averaged to form a composite index (α = 0.81).

##### PANAS-X

A self-report affect scale (PANAS-X; Watson et al., 1988) was used to assess the affective consequences of the goal framing and restriction conditions. This scale consists of 20 words and phrases that describe different positive, negative and neutral feelings and emotions. The participants had to indicate to what extent “*they felt this way right now.*” Scores were averaged to form a composite index for positive (six items; α = 0.84) and negative (nine items) mood (α = 0.78).

All items were presented with a 10-point scale reaching from 1 (not at all) to 10 (absolutely).

### Results

We ran a 2 (goal frame: approach versus avoidance) × 2 (restriction: present versus absent) between-subjects multivariate analysis of variance (MANOVA) on the composite scores of all dependent variables.

#### Reactance

There was a main effect of goal frame, *F*(1,97) = 9.30, *p* = 0.003, η^2^ = 0.09, 1 - β = 0.85 with participants reporting greater experience of reactance in the avoidance (*M* = 3.63, *SD* = 2.29), relative to the approach condition (*M* = 2.73, *SD* = 1.48). This finding confirms our assumption that regardless of any other consideration, individuals would be more reactant when the addition to the program was presented in terms of negative possibilities. In addition, we found a main effect of restriction, confirming that the presence of a real restriction to the participants program in their concentration elicited a greater response in perceived threat when a restriction was present (*M* = 4.17, *SD* = 2.12) than when it was not (*M* = 2.20, *SD* = 0.95), *F*(1,97) = 44.39, *p* < 0.001, η^2^ = 0.31, 1 - β = 1.00. These main effects were qualified by a significant interaction, *F*(1,97) = 5.72, *p* = 0.02, η^2^ = 0.06, 1 - β = 0.66. Means and standard deviations are presented in **Table 2**.

**Table 2:** Means and standard deviations for reactance, agreement, trust, competence, counterarguing, positive affect, and negative affect in Study 2.

		Reactance		Agreement		Trust		Competence		Counter-arguing		Positive Affect		Negative Affect	
Goal frame	Restriction	*M*	*SD*	*M*	*SD*	*M*	*SD*	*M*	*SD*	*M*	*SD*	*M*	*SD*	*M*	*SD*
Approach	Present	3.66	2.05	6.67	2.35	6.11	1.68	6.89	1.63	5.28	2.10	2.27	0.63	1.34	0.40
	Absent	2.11	0.98	7.04	1.68	6.93	1.82	7.13	1.65	4.40	2.37	2.41	0.69	1.35	0.41
Avoidance	Present	5.07	2.28	5.46	1.78	5.16	2.06	5.62	1.86	5.95	2.60	2.14	0.67	1.56	0.46
	Absent	2.59	1.66	5.90	1.93	5.68	2.07	6.11	1.79	5.36	3.07	2.34	0.69	1.54	0.60

#### *Post hoc* Comparisons for the Experience of Reactance

Next, we conducted Bonferroni-corrected *post hoc* comparisons and found that within the approach goal frame conditions, participants showed more reactance in the restriction as compared with the no restriction condition, *p* = 0.013, *d* = 0.92. Similarly, within the avoidance goal frame conditions, participants showed more reactance in the restriction as compared with the no restriction condition, *p* < 0.001, *d* = 1.26. Looked at from a different angle, participants in the freedom restriction conditions showed the most reactance when they were in the avoidance as compared with the approach condition, *p* = 0.005, *d* = 0.69. There was no significant difference between approach and avoidance in the no restriction condition, *p* = 0.553, *d* = 0.33.

#### Message Evaluation, Communicator Evaluation, Cognitive Response, PANAS-X

We found a significant main effect for goal frame on all other dependent variables with the exception of counter-arguing (*p* > 0.1, η^2^ = 0.026; more counter-arguing for avoidance frame) and positive mood (*p* > 0.4, η^2^ = 0.007). There was less agreement with the proposed change (*M*_avoi_ = 5.80, *SD* = 1.75; *M*_appr_ = 6.97, *SD* = 1.74), *F*(1,97) = 10.52, *p* = 0.002, η^2^ = 0.10, 1 - β = 0.89, less perception of communicator competence (*M*_avoi_ = 5.96, *SD* = 1.75; *M*_appr_ = 6.91, *SD* = 1.63), *F*(1,97) = 8.03, *p* = 0.006, η^2^ = 0.08, 1 - β = 0.80, and less trust (*M*_avoi_ = 5.53, *SD* = 1.96; *M*_appr_ = 6.59, *SD* = 1.75), *F*(1,97) = 8.18, *p* = 0.005, η^2^ = 0.08, 1 - β = 0.81, when it was presented with an avoidance frame as compared to an approach frame. Furthermore they experienced more negative mood (*M*_avoi_ = 1.53; *SD* = 0.52, *M*_appr_ = 1.34; *SD* = 0.41) in the avoidance frame condition, *F*(1,97) = 4.61, *p* = 0.034, η^2^ = 0.05, 1 - β = 0.57.

As for restriction, we also found a marginally significant main effect on trust, indicating that people perceived the communicator as less trustworthy when she emphasized the restrictions for the program related to the change (*M* = 5.73; *SD* = 1.90) compared with the absence of a restriction (*M* = 6.45; *SD* = 1.90), *F*(1,97) = 3.79, *p* = 0.054, η^2^ = 0.04, 1 - β = 0.49; see **Table 2** for all means and standard deviations.

#### Mediational Role of Reactance

**FIGURE 2:**
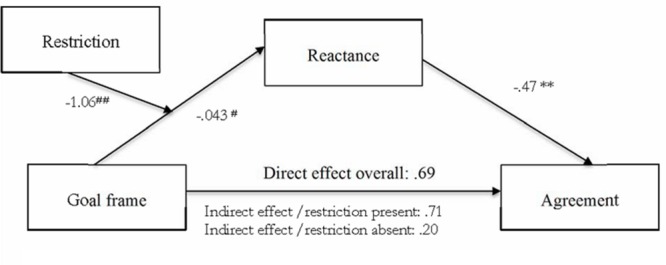
**Moderated mediation model.** All path coefficients are unstandardized regression weights. Total adjusted *R*^2^ for the model = 28, *F*(2,102) = 13.48, *p* < 0.001. ^#^*p* = 0.37, n.s.; ^##^*p* = 0.13, n.s.; ^∗^*p* < 0.05, ^∗∗^*p* < 0.001.

Since the interaction effect of restriction and goal frame suggests that the framing affected reactance only when a restriction was present, we tested the meditational role of reactance here including restriction as a moderator, looking at the behaviorally most relevant outcome variable: agreement. To examine whether the agreement with the proposed change was mediated by the experience of threat to freedom in the form of reactance in dependence of the restriction condition, we conducted a moderated mediation analysis using the PROCESS tool by Hayes (2012). Examining the relationship between the goal frame condition and our central outcome variable ‘agreement,’ bootstrapping techniques employed to test conditional indirect effects confirmed the mediating role of reactance in Study 2 only when an actual restriction was present. The indirect effect of reactance on agreement was significant and positive (0.71) with a 95% confidence interval excluding zero (0.1653 to 1.3147), indicating significant mediation only when a restriction was present: The avoidance frame was associated with more reactance which in turn led to less agreement with the proposed change (see **Figure 2**). However, the interaction term qualifying this mediation did not reach significance (*p* = 0.127)^4^.

#### Inter-correlations of Dependent Variables

Previously, the above six theoretically derived variables have been found to predict decision making following persuasion attempt. To explore these relationships further, correlational analyses were carried out. The majority of the variables correlated significantly with agreement. Most importantly, agreement is negatively associated with reactance *r* = -0.52, *p* < 0.001, while reactance is negatively correlated with both perceived competence, *r* = -0.47, *p* < 0.001, and trustworthiness of communicator *r* = -0.53, *p* < 0.001, and is positively correlated with counterarguing, *r* = 0.60, *p* < 0.001 and negative mood *r* = 0.25, *p* = 0.009. So, the more reactant the person felt the less competent s/he evaluated the communicator. Additionally, the other aspect of how the communicator was perceived (trustworthiness of communicator) and the cognitive response (counterarguing) correlate substantially with both reactance and agreement with proposed change. As predicted, the greater the reactance, the less trustworthy the communicator appears and the more counter-arguing takes place. Conversely, the less trustworthy the participant evaluated the communicator and the more counterarguments s/he elaborated pertaining to the change the lower was her agreement with the message (see **Table 3** for all correlations).

**Table 3:** Study 2: Inter-correlations for reactance, agreement, counterarguing, trust, competence, positive affect, and negative affect.

Composite scores	1	2	3	4	5	6	7
1. Reactance	–	-0.523^∗∗^	0.597^∗∗^	-0.471^∗∗^	-0.528^∗∗^	-0.154	0.253^∗^
2. Agreement		_	-0.415^∗∗^	0.587^∗∗^	0.601^∗∗^	0.180	-0.177
3. Counterarguing			_	-0.502^∗∗^	-0.576^∗∗^	-0.046	0.119
4. Perceived competence				_	0.895^∗∗^	0.246^∗^	-0.235^∗^
5. Trust					_	0.146	-0.310^∗∗^
6. Positive affect						_	-0.009
7. Negative affect							_

*^∗^*p* < 0.05, ^∗∗^*p* < 0.001.*

### Discussion

In Study 2, we replicated the finding that goal frame influences how people respond to a proposed change through the experience of freedom restriction. While a freedom restriction was implied in the change request in Study 1, here we varied the explicit presence or absence of a restriction in a communication that was approach or avoidance framed. Consistent with reactance theory participants’ felt threatened in their freedom when the communication indicated a restriction to their current range of choice for seminars. The restriction, however, led to even more reactance when it was presented in terms of an avoidance frame as compared to an approach frame. So, the presence of an actual restriction played an important role, however, when a restriction was present, the goal frame still significantly affected the degree of reactance. Goal frame, but not restriction, also influenced the evaluation of the communicator such that an avoidance frame led to less positive perceptions of the communicator and worse evaluations of the message. Moreover, the message presented with an avoidance frame as compared with an approach frame led to more counterarguing and less agreement with the proposed change. Looking at the meditational role of reactance here, we find that reactance mediates the impact of the goal frame on agreement with the communicated change only when an explicit freedom restriction was present. When there was no restriction the impact of goal frame was instead mediated, in part, via trust in the communicator, as our explorative mediation analyses suggested (see footnote 4). This suggests, that even messages that do not necessarily restrict the audience may yield disagreement elicited by reduced trust in the communicator when an avoidance goal is the frame.

Specifically, the above findings replicate and extend those reported in a meta-analysis conducted by Rains (2013), where counter-arguing and negative affective responses played a strong direct role in explaining the emergence of reactance. The present study extends these findings by relating the experience of a threat to freedom to goal framing.

## General Discussion

In two experiments, when restricting a person’s freedom avoidance goal frames were associated with more resistance to change on different levels of experience (reactance, performance, communication) and across different domains (achievement, person perception) than approach goal frames. Consistent with reactance theory, participants showed an especially aversive motivational state when a change was phrased in terms of a prohibition. The particular association between psychological reactance and goal frame in explaining the reception of a proposed change suggests that in some circumstances change can be experienced as more of a threat to freedom and therefore as a greater self-threat, particularly when it is frame as an avoidance goal.

Understanding change as a potential threat to self, contingent on the motivational and the situational context, emphasizes the complexity of the psychological processes that are involved. The appraisal of change is not limited to cognitive restructuring and integrating of new information or behavioral scripts. It can also be experienced as a self-relevant message that requires accommodation not only on the behavioral level, but that impacts an individual’s sense of self-determination. As such, the goal-framing of change has implications for theory and practice.

### Theoretical Implications

Extending reactance theory, the present research differentiated the induction of reactance. Across two experiments we differentiated between two outcomes of social pressure: We induced threat to freedom by requesting a person to change strategy (“you must,” Study 1) or by suggesting that a change would lead to a restriction in choice options (“limited access to seminars,” Study 2), while varying the goal frame, with the assumption that avoidance frames imply freedom restriction and may thus increase reactance. Drawing on one part of the underlying axioms of reactance theory, namely, on the effect of impositions and prohibitions (see Wicklund, 1974), leads to the assumption that both the change requests and the restriction of choice may be perceived as threats to freedom and should hence, induce similar amounts of reactance.

The present findings, however, show that a restriction, when presented with an avoidance goal frame is perceived to be more threatening. To our knowledge, no other study has yet examined these specific message features in their implication for important every day decisions on how to phrase a request for change. In communicating change that implies a freedom restriction, emphasis on whether the change is associated with an additional positive outcome or whether a negative outcome can be avoided has a direct effect on the extent to which it is perceived as a restriction to personal freedom: Additional positive outcomes suggest a choice for the individual to decide, if these are wanted, or if the status quo is satisfying enough. Potential negative outcomes put the status quo at risk, which suggests more need to change an existing course of action. Our findings suggest that it is *partly* the implied restriction of freedom conveyed through goal framing that can make an actual freedom restriction to be responded to more or less in terms of a threat to self – here: expressed reactance and the associated variables (impaired performance, negative evaluation of a communicator, disagreement with message).

This interpretation of our findings resonates well with two prominent theoretical accounts. First, it draws on research that demonstrates on how the framing of a goal corresponds to the way in which the goal is represented in memory (Elliot and Friedman, 2007). The negative and undesirable dimension appears to be more salient in memory. Cognitive representations or schemas, when made salient or primed, are more easily retrieved and the likelihood that the forbidden option is selected or undesirable behavior is shown increases. Research in education and health (e.g., Granpre et al., 2003), focus on the question on when children and young adults react most favorably to anti-smoking, anti-drinking or anti-drug campaigns. These findings are supportive of the idea that it is a more adaptive strategy for teachers and health counselors to offer and name a desirable outcome than to focus on the undesirable outcome. Also, research conducted in the context of drug abuse prevention, points at the potential controlling nature of warnings and prohibitions (see, self-determination theory, Ryan and Deci, 2000) and contends that warning of negative outcomes may increase perceived threat to self and thus impair self-regulation. Therefore, instead of communicating prohibitions that are perceived to be controlling, phrasing a request in a manner that enables the individual to seek a solution in a more autonomous way allows for an easier integration of the change into a self-relevant course of action.

Second, it taps into the work by Higgins (1998) on the regulatory focus, which contends that not only chronic states, but also momentary situations – such as induced by message framing – can temporarily yield either a promotion or a prevention focus. For example, feedback messages or task instructions can communicate gain/non-gain information (promotion focus) or non-loss/loss information (prevention focus). Despite the seemingly close affinity between the concepts of approach-avoidance goal frames and self-regulatory focus, these two concepts are theoretically different. The approach-avoidance distinction is rooted in the hedonic principle that contends that individuals strive to attain pleasurable and to stay away from painful outcomes. Self-regulatory focus theory distinguishes between two kinds of goal attainment that vary in chronic focus: attainment of aspirations and accomplishments (promotion focus) and attainment of responsibilities and safety (prevention focus). In combining both theoretical concepts, the approach-avoidance distinction could be viewed as a unifying conceptual thread used to organize and integrate various levels of investigation as it is applicable to dispositional, domain-specific, and situation-specific levels of analysis. For example, work by Higgins (1998) tested whether participants’ motivation for approach versus avoidance was influenced by their regulatory focus and found that participants primed with promotion focus ideals recalled situations better in which they had to approach a match to a goal. The reverse applied to participants primed with a prevention focus.

Across both studies, the present findings show that the type of goal frame directly influences the outcome: When a change message was framed in terms of positive outcomes it led to less reactance, which in turn, yielded a lower (self-perceived) task difficulty, performance or person perception. The explaining role of reactance when a restriction to freedom was implied is consistent with recent findings by Reinhart et al. (2007), who found that gain-framed messages produce more positive reactions toward organ and tissue donation, and lower psychological reactance than loss-framed messages. In this research, following freedom restriction, psychological reactance, and perceived manipulative intent were found to mediate the relationship between framing and message reactions.

### Practical Implications and Future Directions

Research has supported the idea that resistance in the context of organizational change is often attributed to the situation specific to a change (e.g., Burke, 2008). Resistance to change comes from experiencing a lack of choice (i.e., the imposition of change) or from being forced to move away from a known state of being and acting (i.e., the deprivation of stability). The attenuation of aversive affect is critical to the success of a change request. It is important to factor in how changes and change requests are communicated. Understanding the importance of how to communicate change, so that people can engage with it constructively is of high relevance in the context of health communication, organizational change, social and economic justice movements, as well as in educational settings. When people see change as an opportunity to improve the status quo rather than a necessity to maintain it, they feel less threatened in their self-determined action and are more likely to integrate the proposed change, as opposed to showing resistance against change to protect an ego-motive. In this context it could be of interest to – in addition to reactance – explore autonomy, which has shown differential relationships with reactance on how influence is interpreted depending on the source of communication (Pavey and Sparks, 2009)– the frame of the message might add to understanding of the complexity of persuasive communication.

Apparently, the pursuit of some types of goals is more depleting than others. One underexplored issue concerns the process that mediates the link between avoidance framed goal pursuit and goal progress. The presented studies show that avoidance goal frames possess a number of features that are detrimental in the process of regulation (see also Elliot and Sheldon, 1997). Future research may need to further address the question whether and why an avoidance framed goal is perceived to be more threatening to self and why prohibitions more than orders seem to elicit more negative affect. Mediational work on avoidance goal frames remains relatively sparse (see Elliot and Thrash, 2002): several processes appear to account for avoidance goal frames effects such as worry, stress generation, and poor goal progress. Reactance promises to be a new and intriguing mediator to study more in detail.

Furthermore, recent work seems to support our findings in that they point at the threatening nature of avoidance goal frames: avoidance as compared with approach goal frames deplete self-regulatory resources (Oertig et al., 2013). In the context of this research, importantly, the degree of threat was measured directly and compared between the two different goal frame conditions, a measure that the recent work has failed to assess. Therefore, it is only speculative if avoidance versus approach goal frames are experienced as more threatening in our research herein, but it is reasonable to argue so. Additionally, the cumulative findings of two studies appear to be consistent on the notion of the avoidance–reactance–compliance with change link and yield first empirical evidence that the focus on a negative outcome can lead to a more pronounced perceived threat of the self-motive freedom. This threatened self-motive then negatively affects the compliance with the change. Reduced levels of persistence in the avoidance as compared with the approach condition may be an alternative account, which would be worth investigating in future research.

Finally, future research may investigate the process behind the link between avoidance and the emergence of reactance. It is possible that a request to change is perceived to be less legitimate when negative instead of positive outcomes are emphasized (Elliot and Friedman, 2007).

To further enhance the generalizability of our findings, future research may need to implement more distinct state-and trait measures and different time frames. Previous work has discussed state and trait levels of avoidance goal frames (Fryer and Elliot, 2007) and state and trait levels of reactance (Dowd et al., 1991; Shoham et al., 2004).

In sum, given the negative implications of avoidance goal pursuit, a practical approach in social interactions would rely on a message framing that shifts the individual toward the pursuit of approach framed goals. Communicators might be well-advised to avoid high threat messages. Resistance in the context of organizational change is often attributed to the situation specific to the change at hand. Each request to change holds an implicit threat and may thwart the person’s motive of need for control. The thwarted need, in turn, may lead to more resistance to follow the request (Rothbaum et al., 1982). One method to provide control for those whose need was thwarted may consist in attempting to predict events in order to avoid disappointment even if it means a worse outcome compared to the status quo. According to Shelly Taylor’s prominent work on adjustment to threatening events, a person’s thwarted need of control following a change request may be restored in providing meaning in the (loss) experience and providing an opportunity to regain mastery over the event and the possibility to enhance one’s self-esteem (Taylor, 1983).

## Conclusion

The present research shows that communication style in terms of goal frame influences how a request to change is perceived via the experience of threat to freedom. It therefore allows theoretical insights into the process underlying the impairing effect of avoidance goal frames and helps to further our understanding on when and why a request leads to compliance. Understanding more specifically how the frame of a communicated change can affect self-related notions of freedom should ideally help to circumvent blind resistance in communication while allowing to focus on the actual implications of the change.

## Author Contributions

For the present manuscript, DK and VG have contributed equally to study design, data collection, data analysis, interpretation and the theoretical integration of the research. JF has contributed to the Study design and data collection of Study 1, DF has contributed to the conduction of Study 2 and the theoretical integration of the research. All authors have contributed to the preparation of the manuscript with DK and VG taking the lead. All authors have approved the manuscript and take responsibility for all aspects of the work.

## Conflict of Interest Statement

The authors declare that the research was conducted in the absence of any commercial or financial relationships that could be construed as a potential conflict of interest.

## Footnotes

1 An image of each puzzle option is displayed in the section for Supplementary Materials.

2 Running correlations for each goal framing condition separately, however, the approach condition did not reveal a significant association between reactance and task difficulty in the approach condition (*p* ≥ 0.25) supporting the moderated regression analysis. However, in the avoidance condition, reactance is positively correlated with task-difficulty (*r* = 0.46, *p* = 0.023) suggesting that when more threat was underway – as induced by the avoidance framing of the change request-, more experience of threat to freedom was associated with a greater sense of challenge.

3 When including task difficulty as mediating variable the overall model did not reach significance, *R*^2^ = 0.06, *F*(2,50) = 1.58, *p* = 0.217, indicating that the effect of goal frames on performance cannot be explained with a greater perceived task difficulty in the avoidance (*M* = 4.13, *SD* = 1.45) as compared with the approach condition (*M* = 4.07, *SD* = 1.44), *t*(51) = -0.141, *p* = 889.

4 Finding the goal frame affecting agreement via reactance only when a restriction was present on the one hand, and the main effect for goal frame on agreement on the other hand, suggest that framing has the same effect on agreement in the presence or absence of a restriction, however, for different reasons. One candidate mechanism to mediate the effect of goal frame on agreement in the *absence* of a restriction could be trust, since it represents the only other variable besides reactance to be affected by both factors (goal frame and restriction). In order to explore the meditational role of trust on reactance, we conducted a moderated mediation analysis with restriction as a moderator. Examining the relationship between the goal frame condition and agreement, bootstrapping techniques employed to test conditional indirect effects also here suggest a mediating role of trust in Study 2 only when there was no actual restriction. The indirect effect of trust on agreement was significant and negative (-0.66) with a 95% confidence interval excluding zero (-1.1653 to 0.1359), indicating mediation when a restriction was absent: The avoidance frame was associated with less trust which in turn led more reactance. However, this conditional effect was only slightly greater than the non-significant indirect effect of trust on reactance when there was a restriction (-0.50; confidence interval including zero -1.3681 to 0.0529), which is reflected in the fact that here the interaction term qualifying this mediation did not become significant (*p* > 0.6).
