# Supplementary material for: Threat to Freedom and the Detrimental Effect of Avoidance Goal Frames: Reactance as a Mediating Variable
Source: Front Psychol. 2016 May 18;7:632. doi: 10.3389/fpsyg.2016.00632 (PMC4870279; doi:10.3389/fpsyg.2016.00632)
Supplement: Supplementary file 1 [file Data_Sheet_1.PDF]

Supplemental materials: Figure 2 b.

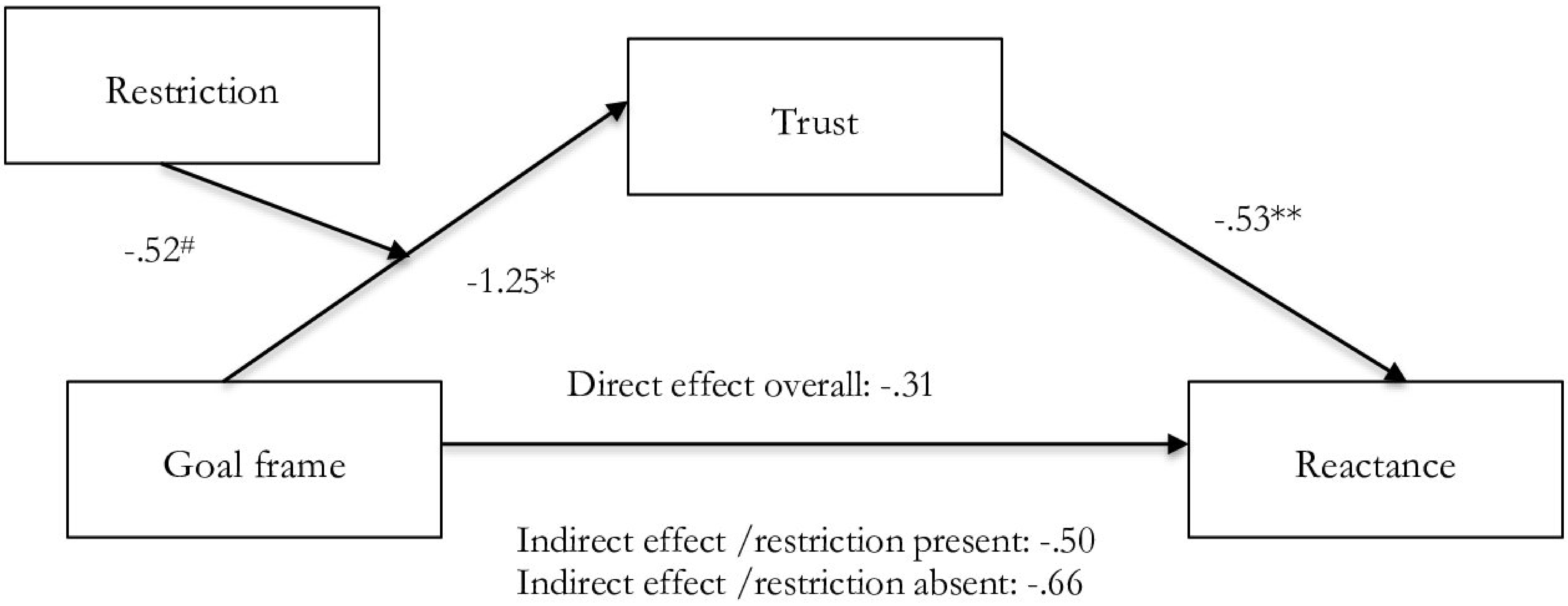

Figure 2 c: Moderated mediation model. All path coefficients are unstandardized regression weights. Total adjusted R^2 for the model = .28, F (2, 100)= 19.86, p <.001.
# p = .35, n.s.; * p <.05; ** p <.001
