# Supplementary material for: Threat to Freedom and the Detrimental Effect of Avoidance Goal Frames: Reactance as a Mediating Variable
Source: Front Psychol. 2016 May 18;7:632. doi: 10.3389/fpsyg.2016.00632 (PMC4870279; doi:10.3389/fpsyg.2016.00632)
Supplement: Supplementary file 2 [file Data_Sheet_2.PDF]

Supplemental materials

Figure 3 a. Task of Study 1: Searching for the letter p in entirely displayed box (among the letters d, p, q, b, r, g; 114 of each letter in entire box).

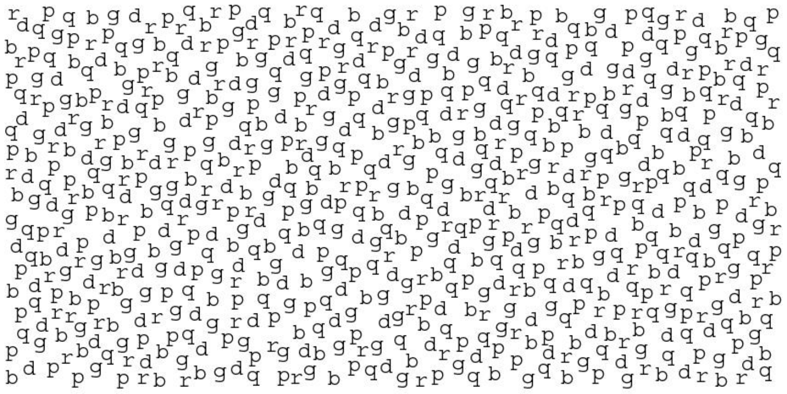

Figure 3 b. Task of Study 1: Searching for the letter p column by column (among the letters d, p, q, b, r, g; 114 of each letter in entire box).

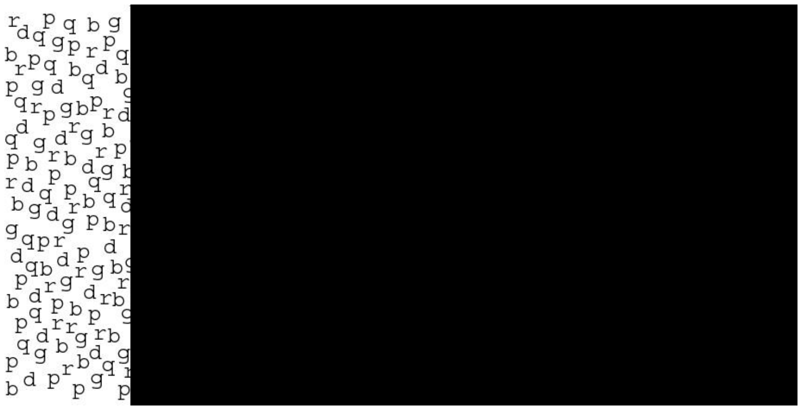

Figure 3 c. Task of Study 1: Searching for the letter p row by row (among the letters d, p, q, b, r, g; 114 of each letter in entire box).

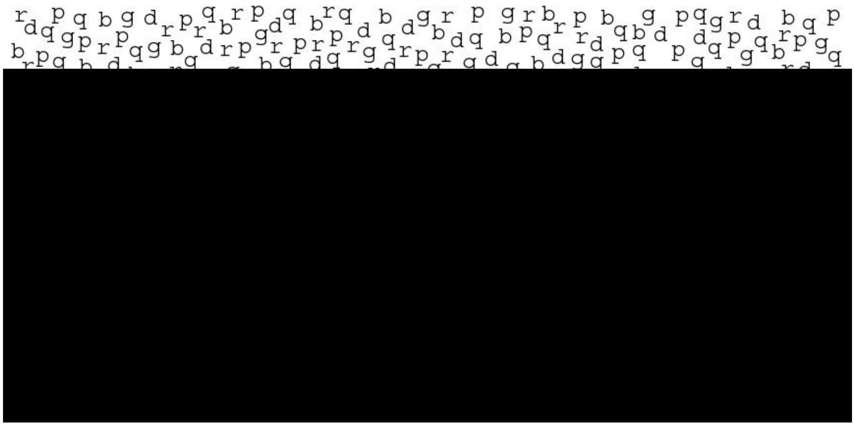

Figure 3 d. Task of Study 1: Searching for the letter p grid by grid (among the letters d, p, q, b, r, g; 114 of each letter in entire box).

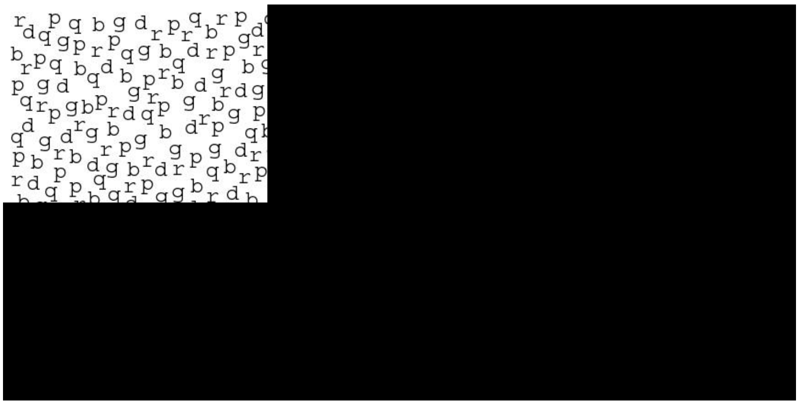

1. **Puzzle 1 accuracy (before change was imposed) at round 1**

Zwischensubjektfaktoren

		Wertbeschriftung	H
puzzle 1, option chosen	1,00	entire puzzle	1
	2,00	columns	22
	3,00	rows	20
	4,00	grids	10

Deskriptive Statistiken

Abhängige Variable: puzzle 1 accuracy

puzzle 1, option chosen	Mittelwert	Standardabweichung	H
entire puzzle	20,0000	.	1
columns	5,7727	5,31782	22
rows	7,3000	5,95686	20
grids	12,6000	7,10555	10
Gesamtsumme	7,9057	6,49930	53

Tests der Zwischensubjekteffekte

Abhängige Variable: puzzle 1 accuracy

Quelle	Typ III Quadratsumme	df	Quadratischer Mittelwert	F	Sig.	Partielles Eta hoch zwei
Korrigiertes Modell	474,065[a]	3	158,022	4,495	,007	,216
Konstanter Term	1744,941	1	1744,941	49,639	,000	,503
p1option	474,065	3	158,022	4,495	,007	,216
Fehler	1722,464	49	35,152			
Gesamtsumme	5509,000	53				
Korrigierter Gesamtwert	2196,528	52				

a. R-Quadrat = ,216 (Angepasstes R-Quadrat = ,168)

2. **Puzzle 2 accuracy (after change was imposed) at round 2**

Zwischensubjektfaktoren

		Wertbeschriftung	H
puzzle 2, option chosen	1,00	entire puzzle	4
	2,00	columns	16
	3,00	rows	16
	4,00	grids	17

Deskriptive Statistiken

Abhängige Variable: puzzle 2 accuracy

puzzle 2, option chosen	Mittelwert	Standardabweichung	H
entire puzzle	8,7500	12,50000	4
columns	6,9375	6,24466	16
rows	5,6875	5,68880	16
grids	8,0588	9,07242	17
Gesamtsumme	7,0566	7,48695	53

Tests der Zwischensubjekteffekte

Abhängige Variable: puzzle 2 accuracy

Quelle	Typ III Quadratsumme	df	Quadratischer Mittelwert	F	Sig.	Partielles Eta hoch zwei
Korrigiertes Modell	58,764[a]	3	19,588	,336	,799	,020
Konstanter Term	1997,010	1	1997,010	34,262	,000	,411
p2option	58,764	3	19,588	,336	,799	,020
Fehler	2856,066	49	58,287			
Gesamtsumme	5554,000	53				
Korrigierter Gesamtwert	2914,830	52				

a. R-Quadrat = ,020 (Angepasstes R-Quadrat = -,040)
